# Low-level alternative tRNA priming of reverse transcription of HIV-1 and SIV in vivo

**DOI:** 10.1186/s12977-019-0473-2

**Published:** 2019-04-04

**Authors:** Christine M. Fennessey, Celine Camus, Taina T. Immonen, Carolyn Reid, Frank Maldarelli, Jeffrey D. Lifson, Brandon F. Keele

**Affiliations:** 1 AIDS and Cancer Virus Program, Frederick National Laboratory for Cancer Research, Frederick, MD 21702 USA; 2 HIV Dynamics and Replication Program, National Cancer Institute, Frederick, MD 21702 USA

**Keywords:** SIV, HIV, Primer binding site, tRNA, Next-generation deep-sequencing

## Abstract

**Background:**

Reverse transcription (RT) of HIV and SIV is initiated by the binding of the acceptor stem of tRNA^Lys3^ to the primer binding site (PBS) of the viral RNA genome. Previous studies have suggested that this tRNA^Lys3^ is not the only molecule capable of priming reverse transcription, and that at least one other lysyl tRNA, tRNA^Lys5^, which has an acceptor stem sequence varying from tRNA^Lys3^ by only a single transition mutation resulting in the integration of a thymine (T) at position 8 of the PBS in the viral genome, can prime reverse transcription.

**Results:**

We undertook an unbiased approach, evaluating the primer binding site by deep-sequencing of HIV and SIV directly from the plasma of 15 humans and 11 macaques. We found that in humans there are low but measurable levels of viral RNA genomes harboring a PBS containing the noncanonical T at position 8 (PBS-Lys5) corresponding to the tRNA^lys5^ sequence and representing an average of 0.52% (range 0.07–1.6%) of the total viral population. This value is remarkably consistent with the proportion of PBS-Lys5 we identified in a cross-sectional assessment of the LANL HIV database (0.51%). In macaques chronically infected with SIVmac239, the PBS-Lys5 was also detected but at a frequency 1-log less than seen for HIV, with an average of 0.056% (range 0.01–0.09%). At this proportion, PBS-Lys5 was comparable to other transition mutations, making it impossible to determine whether the mutation observed is a result of use of tRNA^Lys5^ as an RT primer at very low levels or merely the product of in vitro cDNA synthesis/PCR error. We also identified two novel PBS sequences in HIV and SIV at low levels in vivo corresponding to tRNA^Lys6^ and tRNA^Lys1,2^, suggesting that these tRNAs may rarely also be used to prime RT. In vivo reversion of the PBS-Lys5 found in SIVmac239 was rapid and reached background levels by 30 days post-infection.

**Conclusions:**

We conclude that while alternative tRNAs can initiate reverse transcription of HIV and SIV in vivo, their overall contributions to the replicating viral population are small.

**Electronic supplementary material:**

The online version of this article (10.1186/s12977-019-0473-2) contains supplementary material, which is available to authorized users.

## Background

SIVmac239 is a lab-adapted strain of SIV frequently used both in vitro and in vivo in rhesus macaque studies to model aspects of HIV biology. However, despite its frequent usage, Alexander et al. [1] identified four suboptimal nucleotides in the genome of this viral clone which are likely the result of lab-generated errors. These four nucleotides are found in the Pol (1129 and 2339) and Env (2251) genes and within the primer binding site (PBS) (position 8 of the PBS). Although the time to reversion of these suboptimal sites is variable, each presumably has a fitness cost that may alter the dynamics of primary infection leading to increased variability based on when the correction is made. The most interesting of these mutations is that of the PBS since the sequence of the integrated viral PBS is determined by the sequence of the acceptor stem of the tRNA used to initiate reverse transcription (RT) and is made by copying RNA in both plus and minus strands. Mutations at other sites are generated during the error-prone process of reverse transcription.

It has been known for nearly 50 years that the exact sequence of the PBS determines the specific tRNA that can bind and/or the affinity of the tRNA to the PBS [2–4]. HIV and SIV both preferentially bind tRNA^Lys3^ to initiate RT, as the acceptor stem of the tRNA molecule is perfectly complementary to the PBS of both HIV and SIV (PBS-Lys3) [5–8]. Mutations in the PBS sequence could therefore lead to less efficient binding of the canonical tRNA^Lys3^, potentially affecting reverse transcription and viral replication [9–11]. The cytosine (C) to thymine (T) point mutation in the SIVmac239 PBS identified by Alexander et al. reverts to the canonical C, suggesting that alternate tRNAs such as tRNA^Lys5^, which is fully complementary to the suboptimal PBS-Lys5 do not function well or are insufficiently expressed in host cells [3, 9]. Indeed, Soderberg et al. [12] describe reversion of this nucleotide in an SIVmac239 derivative in vitro and report drastically increased replicative fitness following this reversion.

In our previous study [13] we showed that the PBS mutation is characterized by rapid reversion kinetics during replication in cell lines. The SIVmac239 virus was grown in vitro in SupT1-R5 cells for 2 months and the proportions of PBS-Lys3 and PBS-Lys5 were analyzed by single genome amplification (SGA). Sequence analysis of cultures demonstrated complete reversion to PBS-Lys3 by day 21, at an average rate of 5% of the population reverting per day. We showed that such rapid reversion occurs even in a single cycle infection experiment with only a single RT event and posit that the usage of tRNA^Lys3^ to prime RT, in conjunction with host mismatch repair mechanisms and/or premature stops in transcription, leads to reversion of the suboptimal T at position 8 of the PBS to its canonical C.

Immediately following this publication, Berkhout et al. [14] published an opinion piece that suggested that the presence of the non-canonical T at position 8 of the PBS sequence is not the result of in vitro cDNA synthesis/PCR error, but rather was explained by the use of tRNA^Lys5^ as an alternate primer for the initiation of reverse transcription. They proposed that tRNA^Lys3^ and tRNA^Lys5^ anneal the suboptimal PBS sequence with the same efficiency and that subsequent cell division will result in the production of proviruses with both suboptimal and optimal PBS sequences. It has been reported that tRNA^Lys5^ is a minor variant of tRNA^Lys^ representing only 10% of the total tRNA^Lys^ found in the human cell line SupT1 [15]. Due to the relative abundance of tRNA^Lys3^ and tRNA^Lys5^, they hypothesize that regardless of the initial PBS sequence, cell division and subsequent rounds of replication will lead to a fixed 95:5 ratio of PBS-Lys3 to PBS-Lys5 sequences [14, 15].

Operating under the idea that the PBS sequence is reflective of the tRNA used to prime reverse transcription, we sought to obtain an unbiased view of alternate tRNA use in reverse transcription. We therefore performed next-generation deep-sequencing to analyze the PBS from plasma virus collected from both humans and rhesus macaques. Examination of PBS sequences from chronically infected humans revealed a small population of sequences complementary to the tRNA^Lys5^ but at levels much lower than 5%. When similar analyses were conducted on plasma samples from chronically infected rhesus macaques, we found that the level of PBS corresponding to tRNA^Lys5^ fell into the range of RT and PCR error, making it impossible to conclusively define a role for this tRNA in SIV reverse transcription. The differences in these relative proportions may indicate differential expression of tRNA in humans and macaques, which could lead to variation in the tRNA priming of reverse transcription in these two species. Interestingly, we also identify two novel sequences corresponding to tRNAs not previously reported to have a role in reverse transcription in vivo: tRNA^Lys6^ and tRNA^Lys1,2^. Finally, we report that in macaques, the transition of the PBS-Lys5 sequence to PBS-Lys3 occurs quickly during the acute phase of infection and is at background levels within 30 days of infection.

## Results

### Analysis of PBS-Lys5 in chronic HIV infection

**Fig. 1:**
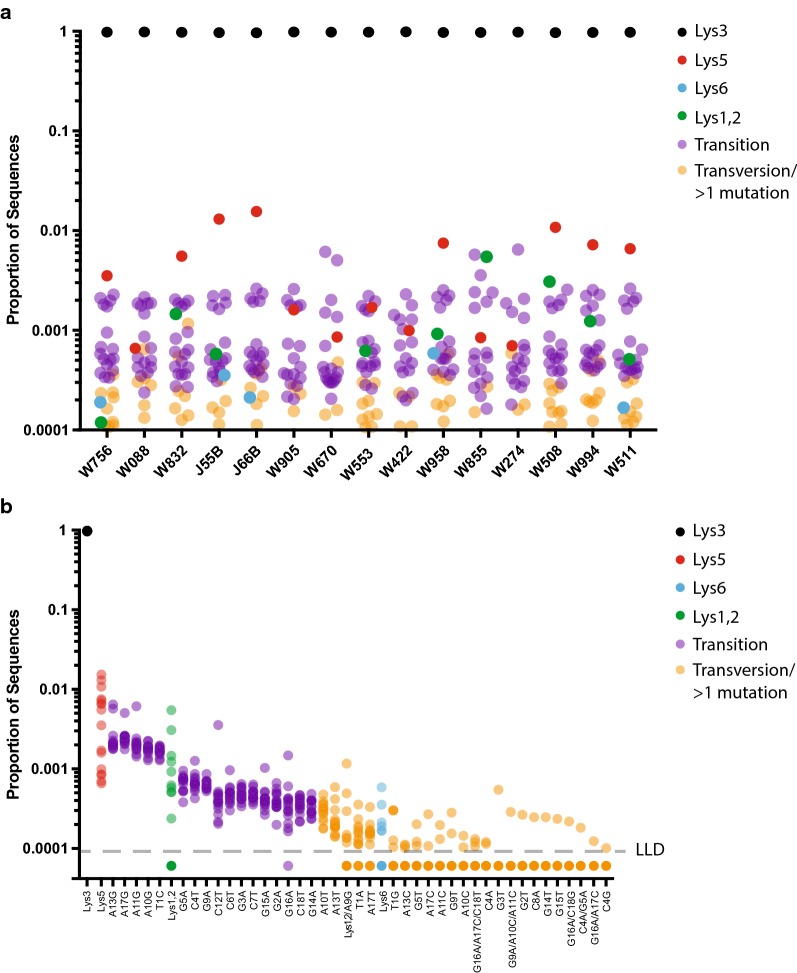
Relative proportions of HIV PBS-Lys3 (black), PBS-Lys5 (red), PBS-Lys6 (cyan), PBS-Lys1,2 (green), all transition mutations (purple), and all transversion mutants or sequences with greater than 1 mutation (orange) sequences obtained using next-generation sequencing. Results are displayed by individuals (**a**), or as mutations rank ordered based on the average proportion in all individuals (**b**)

In an effort to better understand the role of different tRNAs in reverse transcription in humans, we performed an unbiased, deep-sequencing analysis of the primer binding site sequences detectable in plasma HIV samples. Plasma samples from 15 HIV-infected but cART untreated individuals were collected and analyzed. An illumina based sequencing protocol was developed to specifically amplify and sequence the PBS region of the HIV genome. Sequencing was performed on a MiSeq instrument and the proportion of each unique PBS sequence was determined. The PBS sequence that results in perfect complementarity to tRNA^Lys3^ (PBS-Lys3), the canonical tRNA molecule used for HIV/SIV reverse transcription, represented almost the entire population of the sample, with values ranging from 96.5 to 98.5% of all sequences analyzed per individual. 45 other sequence variants were detected using this approach, including all 18 transition mutations and 18 transversion mutations, as well as several sequences that contained more than one mutation (Fig. 1). Among the mutations detected was the sequence that corresponds to tRNA^Lys5^ (PBS-Lys5), a molecule that has been suggested to also act as a primer for HIV/SIV reverse transcription and cause the generation of PBS-Lys5 in progeny virus [3, 15]. The abundance of this sequence encompassed greater than a l-log range across the different infected individuals studied, from 0.066 to 1.6% with an average of 0.52%. The distribution of the PBS-Lys5 virus from 8 of 15 individuals revealed PBS-Lys5 as the second most frequent sequence within the viral population with measured values higher (0.35–1.6%, average 0.88%) than the remaining 7 individuals (0.066–0.17%, average 0.11%) whose the PBS-Lys5 sequence is not the second most frequent sequence. The measured values for the PBS-Lys5 from these 7 individuals fell well within the range of values for other transition mutations, which ranged from 0.016 to 0.65% (average: 0.1%). These data indicate that at least in some individuals, PBS-Lys5 can occur in vivo and is not likely to represent only RT/PCR error. Interestingly, the average measured value of PBS-Lys5 in these 15 individuals (0.52%) falls 1-log below the 5% level that Das and Berkhout predict would be the observed in humans [14, 15].

### Analysis of PBS-Lys5 in chronic SIV infection

**Fig. 2:**
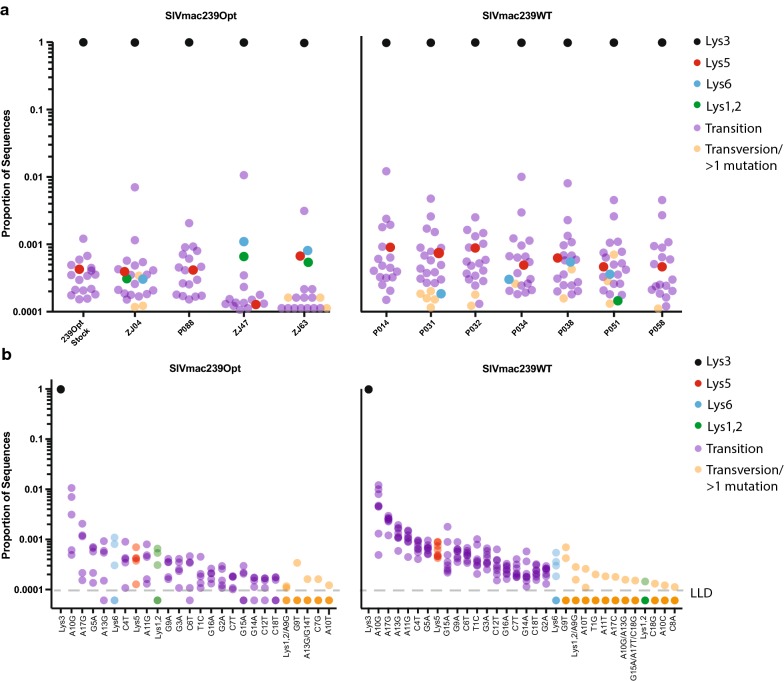
Relative proportions of SIV PBS-Lys3 (black), PBS-Lys5 (red), PBS-Lys6 (cyan), PBS-Lys1,2 (green), all transition mutations (purple), and all transversion mutants or sequences with greater than 1 mutation (orange) sequences obtained using next-generation sequencing. Results are displayed for the SIVmac239Opt infectious stock as well as individual animals infected with either SIVmac239 or SIVmac239Opt (**a**), or as mutations rank ordered based on the average proportion in all animals infected with the same viruses (**b**)

To characterize the PBS sequences in chronically infected rhesus macaques, we performed a similar next-generation deep-sequencing approach with SIV-specific primers on plasma samples collected from 11 animals, 7 of which were infected with wild-type SIVmac239 and the remaining 4 infected with SIVmac239Opt. The wild-type SIVmac239 clone contains the PBS-Lys5 sequence and the SIVmac239Opt clone contains the PBS-Lys3 sequence as well as the corrected version of the 3 other suboptimal mutations in Pol (1129 and 2339) and Env (2251). Across all 11 animals, we found that the vast majority of all of viral sequences consisted of virus harboring the PBS-Lys3 (97.25–99.31%). As with the HIV sequences, all 18 transition mutations were identified, as well as 9 transversion mutations (Fig. 2). While PBS-Lys5 was detected in animals, it was detected at very low levels ranging from 0.013 to 0.090% (average: 0.056%), and in none of the animals was it one of the top 3 most frequent PBS sequences. By comparison, the average measured value of the other 17 PBS transition mutations was 0.086% (Fig. 2b). These data suggest that in macaques, the use of tRNA^Lys5^ is either nonexistent or below the level of in vitro cDNA synthesis/PCR error.

### Analysis of Novel PBS sequences

**Fig. 3:**
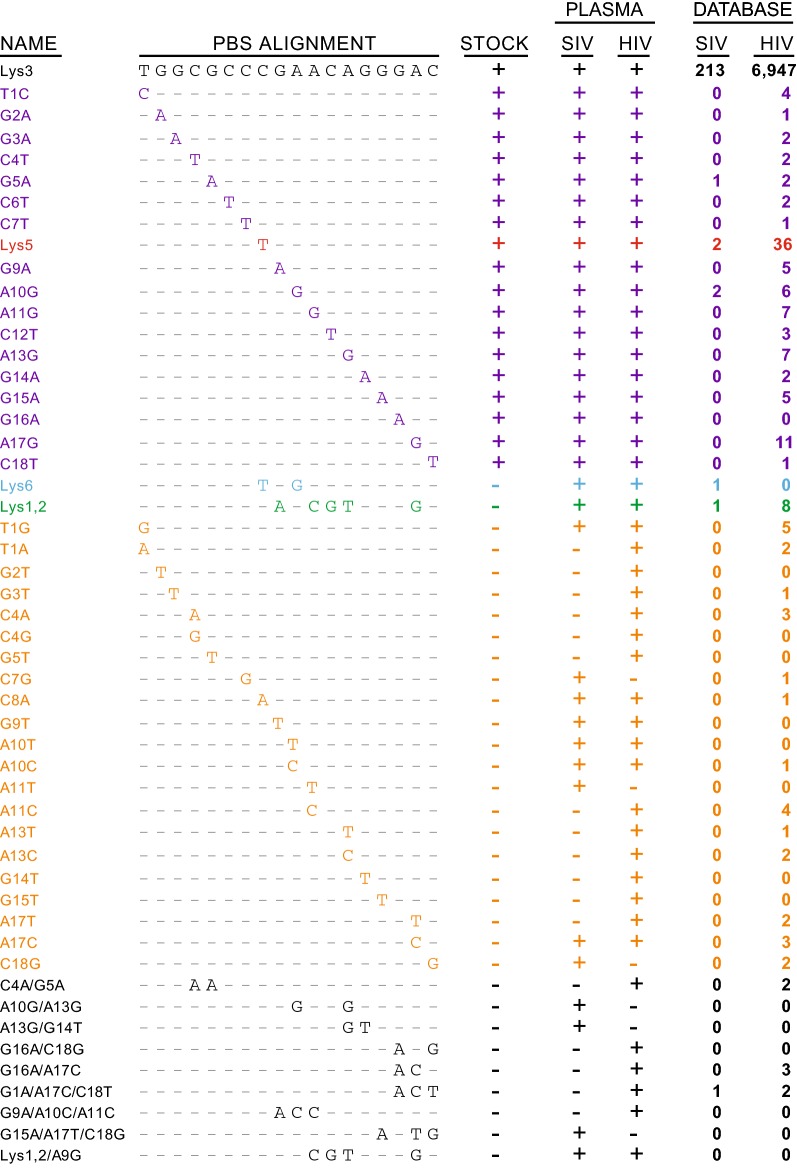
Alignment of all detected sequences found in the HIV and SIV samples and the SIVmac239Opt infectious stock, highlighting the nucleotides that deviate from the canonical PBS-Lys3 sequence. For the stock and plasma samples, the +/− indicates whether the particular sequence was found in that sample set. For the database, the number listed indicates how many of each sequence were identified in the LANL HIV database in SIV or HIV sequences

In addition to all 18 transition mutations and 21 total transversion mutations detected in the PBS region of both HIV and SIV, 11 other PBS sequences containing 2–5 point mutations were detected (Fig. 3). These sequences are interesting because they represent sequences in which either the low-probability event of multiple point mutations in a short region occurred, or they are due to alternate tRNA priming. None of these sequences were found in the SIVmac239Opt infectious stock, implying that these mutations were not commonly generated during PCR and therefore are likely real, in vivo generated mutations. However, the majority (9 of 11) of these sequences are found at very low frequencies and only in a few humans or macaques. The other 2 mutations correspond to the acceptor stem of 2 known tRNAs: tRNA^Lys6^, which contains 2 mutations from the canonical PBS-Lys3 sequences (C8T and A10G) and tRNA^Lys1,2^, which are 5 mutations from the canonical PBS-Lys3 sequence (G9A, A11C, C12G, A13T, and A17G). Interestingly, tRNA^Lys1,2^ are lysine tRNAs with identical acceptor stems and with a CTT anticodon (as opposed to the TTT anticodon on the Lys3, Lys5, and Lys6 tRNAs). The PBS-Lys6 sequence was found in 5 of 15 humans with a mean proportion of 0.0003 and in 7 of 11 macaques with a mean proportion of 0.0005. The PBS-Lys1,2 sequence was found in 9 of 15 humans with a mean proportion of 0.015 and in 4 of 11 macaques with a mean proportion of 0.0004.

To test if the sequences matching tRNA^Lys^^6^ or tRNA^Lys^^1,2^ were real or due to PCR error, we evaluated all sequences with more than one mutation away from a known tRNA. We observed 5 such sequences across all 15 tested humans with a mean proportion of 0.00019 and 3 such sequences in macaques with a mean proportion of 0.00016 (Figs. 1b, 2b). Importantly, each of these mutations was unique and found in only one human or macaque. Given the very low error rate of single transition or transversion mutations and the infrequency of multi-site mutations, we conclude that the mutations matching tRNA^Lys^^6^ and tRNA^Lys^^1,2^ are real and arose through the priming of alternate tRNAs rather than in vitro cDNA synthesis or PCR error.

### Longitudinal Analysis of PBS-Lys5 Persistence

**Fig. 4:**
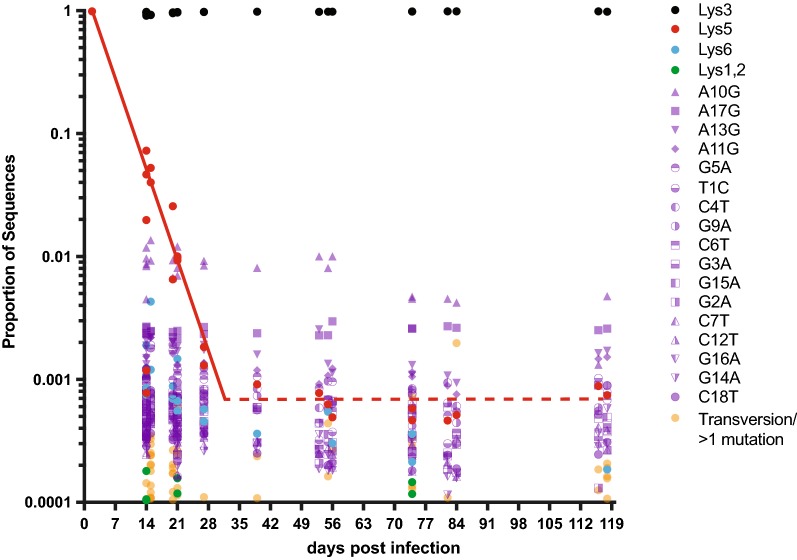
Relative proportions of PBS sequences resulting from deep-sequencing of rhesus macaques acutely infected with SIVmac239 over the course of 120 days. PBS-Lys3 (black), PBS-Lys5 (red), PBS-Lys6 (cyan), PBS-Lys1,2 (green), individual transition mutants (purple), and transversion mutants/sequences with greater than 1 mutation (orange) are shown. The solid red line depicts the linear regression of PBS-Lys5 until steady state; the dashed red line indicates the average detectable level of PBS-Lys5 once steady state has been reached

In our previous publication, we performed an in vitro experiment in which SupT1-CCR5 cultures were infected with SIVmac239, and the PBS sequence of the culture supernatant analyzed by single genome amplification over time. We reported on the rapid reversion of the PBS-Lys5 in SIVmac239 to PBS-Lys3, such that PBS-Lys5 declined to undetectable levels by day 21 [13]. To assess whether a similar phenomenon occurred in vivo and to increase sampling depth, here we performed next-generation deep-sequencing from plasma virus collected from 8 rhesus macaques intravenously infected with SIVmac239 (Fig. 4). Similar to the in vitro study, rapid reversion of PBS-Lys5 was observed. The frequency of PBS-Lys5 decreased exponentially at a rate of 0.24 per day to a steady-state level of 0.06% at day 30, which was maintained for at least 120 days post infection. All other non-canonical PBS sequences were also analyzed over time, and unlike PBS-Lys5, all of those mutations were maintained at approximately the same level in the population over the course of the study.

### Analysis of the HIV/SIV database

Given the low-level proportion of PBS mutations identified during deep-sequencing of a few individuals and animals, we next sought to assess the frequency of PBS mutations across the HIV and SIV sequence database. We hypothesized that if individuals had polymorphic mutations in PBS, these would be identified in other sequencing studies. We performed a comprehensive, unbiased assessment of the PBS sequences found in the HIV and SIV database of the Los Alamos National Laboratory (HIV.lanl.gov). At the time of our search, 4 SIV sequences corresponding to PBS-Lys5 were found in the database, compared to 216 PBS-Lys3 sequences. Of these SIV PBS-Lys5 sequences, 1 was SIVmac239 itself, 1 was SIVmac251 (the viral swarm from which SIVmac239 was derived), and 2 were subclones derived from SIVmac239. Thus, excluding these 2 SIVmac239 replicates, there are only 2 authentic SIVmac sequences (out of a total of 221) containing the PBS-Lys5, equivalent to 0.9% of the SIV database. This is in contrast to the HIV-1 database, in which we found 36 PBS-Lys5 sequences out of a total of 7086 PBS sequences, equating to 0.51% of the total database. Additionally, for HIV-2, which is genetically similar to SIVmac but isolated from humans, one PBS-Lys5 sequence was identified out a total of 80 PBS sequences (1.3%). Interestingly, we also found PBS sequences corresponding to tRNA^Lys6^ (1 SIVmac sequence) and tRNA^Lys1,2^ (8 HIV sequences from 4 donors), as well as other PBS transition mutations (Additional file 1: Table S1). For HIV infected humans and SIV infected rhesus macaques, these data suggest that at very low levels, noncanonical tRNAs can be used to initiate RT which can produce virus with PBS sequences complementary to the acceptor stem of the priming tRNA.

## Discussion

Previously, we described the generation of an SIVmac239 clone that has 4 suboptimal mutations repaired to the canonical (and presumably optimal) bases (SIVmac239Opt). Reversion of the PBS was rapid in vitro even after a single round of infection. We suggested this reversion occurred when RT was primed by tRNA^Lys3^, despite its imperfect complementarity to wild-type SIVmac239 PBS. If primed with tRNA^Lys3^, resultant integrated viral genomes would contain a mismatch at position 8 in the PBS, which could be resolved by cellular DNA repair mechanisms [13–15]. This study also describes the benefits of using SIVmac239Opt in NHP studies as this model eliminates the uncontrolled variable of suboptimal nucleotide reversion during in vivo studies. It has been suggested that the proportion of the tRNA^Lys^ variants found in the host cells determine the proportion of PBS-Lys5 and PBS-Lys3 viral genomes. Given the estimated proportion of tRNA in human cells, it was proposed that 5% of all progeny virus would contain the PBS-Lys5 sequence regardless of the sequence of the infecting virus [14, 15], although the rate to reach this equilibrium was not estimated.

In order to perform a comprehensive analysis of the PBS sequences found in both HIV infected humans and SIV infected rhesus macaques, we used deep-sequencing and the LANL HIV database to gain insight into the relative abundance of various PBS sequences, taking advantage of the fact that PBS sequences are reflective of the tRNAs used to prime the reverse transcription mechanism. We first examined the sequences of virus collected from 15 HIV-infected individuals. In this population, as expected, the majority of the sequences corresponded to the tRNA^Lys3^ primer, though we also found a low level of virus containing the PBS-Lys5 sequence. However, this suboptimal sequence was only detected in, on average, 0.52% of all sequences, which is 1-log less than estimated previously [14, 15]. Interestingly, we noted a large range in measured PBS-Lys5 values, spanning ~ 1.5-logs which might reflect polymorphisms in tRNA expression between individuals. Furthermore, after searching the LANL database, only 0.51% of all reported HIV sequences including a PBS sequence contained PBS-Lys5. Therefore, we conclude that tRNA^Lys5^, in at least in some individuals, can contribute to the maintenance of a small population of HIV in vivo producing virus progeny with a PBS-Lys5 sequence.

Additionally, plasma samples collected from rhesus macaques chronically infected with either SIVmac239 or SIVmac239Opt were similarly analyzed, and only 0.056% of all sequences contained the PBS-Lys5 sequence. This value is ~ 2-logs less than the 5% predicted to retain the PBS-Lys5 sequence. At this very low level, it was not possible to distinguish PBS-Lys5 sequences as authentic and in vivo derived versus ex vivo generated during cDNA synthesis and PCR. Unlike HIV where over half of tested individuals maintained the PBS-Lys5 sequence as the second most abundant variant of all sequences identified, in SIV infected macaques, in no animal was PBS-Lys5 sequence the second most prevalent mutation. Indeed, in the SIV samples, statistical analysis failed to indicate enrichment of PBS-Lys5 sequence over other transition mutations. Thus, it remains unclear if SIV in rhesus macaques utilizes tRNA^Lys5^ to prime RT, but if so, it is at very low levels and ~ 2-logs less than previously estimated [15].

Since the infectious stock of wild-type SIVmac239 contains the PBS-Lys5 sequence, and we cannot conclude that tRNA^Lys5^ is available in rhesus macaques for priming, we surmise that SIVmac239 binds tRNA^Lys3^ resulting in an integrated genome that contains a mixed base at position 8 of PBS (cytosine on the plus strand and an adenine on the minus strand). If the cytosine is retained during host repair, then progeny virus will be produced with PBS-Lys3. If the minus strand adenine is retained during host repair, then virus will be produced with PBS-Lys5. If no repair is made, progeny virus will also produce virus with PBS-Lys5 [13]. Thus, to determine the rate of reversion to PBS-Lys3, we sequenced the virus in the plasma of macaques intravenously infected with wild-type SIVmac239 during primary infection. We observe a rapid reversion of the preexisting PBS-Lys5 sequence to the PBS-Lys3 sequence. Approximately 50% of sequenced virions had reverted by day 5 post-infection, and by day 30, the PBS-Lys5 levels dropped to the endogenous error level, where they remained for at least 120 days. These data demonstrate the rapid loss of this mutation in wild-type SIVmac239 likely due to host repair or cell division. Given the high replicative capacity of SIVmac239, it appears unlikely that utilizing tRNA^Lys3^ to prime RT in a virus containing a PBS-Lys5 sequence alters its overall dynamics. However, during mucosal infection or at limiting dilution intravenous infection, early correction to PBS-Lys3 in one viral lineage might allow for its preferential amplification over clones that retain the PBS-Lys5 sequence, introducing heterogeneity into viral population dynamics.

In vitro comparisons of wild-type SIVmac239 and SIVmac239Opt revealed SIVmac239Opt to be slightly more efficient at replicating in rhesus CD4+ T cells [13]. Since SIVmac239Opt is corrected at all 4 suboptimal bases and not just the mutation in the PBS, additional work is needed to directly test the binding and replicative differences of tRNA^Lys3^ binding PBS-Lys5 compared to the cognate binding of tRNA^Lys3^ and PBS-Lys3. We also suggest that nonhuman primate studies seeking to elucidate acute infection events using macaques infected with wild-type SIVmac239 may be introducing an uncontrolled experimental variable, depending on the fitness cost of tRNA^Lys3^ binding to PBS-Lys5 sequence.

One of the mechanisms used by retroviruses to ensure replication competency upon infecting target cells is to incorporate the tRNA used to prime reverse transcription into the nascent virus particle. For example, Rous sarcoma virus (RSV) selectively packages tRNA^Trp^, and Moloney murine leukemia virus (Mo-MuLV) packages tRNA^Pro^ (though to a lesser extent) [16]. Similarly, tRNA^Lys^ is selectively packaged into HIV and SIV virus particles via binding of the lysyl-tRNA synthetase protein and the Gag and Gag-Pol proteins [17–19] such that tRNA^Lys^ molecules are enriched approximately tenfold in the virus particle [16]. Furthermore, previous studies demonstrate the presence of tRNA^Lys1,2^, and tRNA^Lys3^ in the viral particle, although other tRNA^Lys^ isotypes have not been directly queried [20, 21]. Others suggest that the binding of the tRNA to the Gag-Pol protein occurs concomitantly with translation, and thus the tRNAs selected are dependent on the local tRNA concentration [22, 23]. Regardless of the mechanism, we show here that the presence of PBS-Lys1,2 sequence in viral genomes corresponds to tRNA^Lys1,2^ being used as primer for RT initiation. Previous studies demonstrate the usage of this tRNA to prime HIV RT in a manipulated in vitro system (suggesting also that the reverse transcriptase is not specific for tRNA^Lys3^) [24, 25], yet here we find evidence for its use in vivo. Whereas the identification of other transition mutations (and indeed also the PBS-Lys5 sequence in macaques) may be attributed to random mutations arising during RT or PCR, it is highly unlikely that the 5 mutations necessary to generate the PBS-Lys1,2 sequence are due to in vitro errors. Additionally, the 2 changes required for PBS-Lys6 to be generated from the canonical PBS are unlikely due to cDNA synthesis or PCR error even though both mutations are transition mutations. These data were confirmed by the identification of PBS-Lys1,2 sequences in the LANL HIV database with 10 PBS-Lys1,2 sequences found in HIV and 1 PBS-Lys6 sequence found in SIV. Interestingly, we also found most of the other transition/transversion mutations detected in our deep-sequencing approach in the LANL database. These mutations do not correspond to the acceptor stem of any tRNAs, Lys or otherwise, and may just be the result of cDNA synthesis or PCR error. Also of interest, although previous studies demonstrate that tRNA^Ile^ and tRNA^Asn^ are also packaged into the HIV particle [26], in our study, we do not find any evidence for the involvement of these tRNAs in RT initiation.

Importantly, we found that the expression of tRNA^Lys5^ in humans is likely sufficient to produce viral progeny with PBS-Lys5 sequence. However, this could not be demonstrated in the macaque system where the measured difference between PBS-Lys5 and background was minimal. The observation that tRNA^Lys5^ may be a factor in a human system but not in macaques leads to an interesting hypothesis on the origin of the PBS suboptimal nucleotide in wild-type SIVmac239. This clone was originally isolated from a rhesus macaque in late stage simian AIDS, followed by passage in the human cell line HUT-78 to generate a viral isolate which was subsequently cloned [27]. It is possible that the availability of tRNA^Lys5^ from the human cell line allowed the introduction of the suboptimal PBS-Lys5 in the virus which was subsequently cloned to produce the SIVmac239 clone. Furthermore, additional work will need to be done to attempt to explain the disparity between the estimated PBS-Lys5 sequence proportion [14, 15] and the proportions determined experimentally. It will be interesting to determine the availability of noncanonical tRNAs in various experimental and natural hosts of HIV and SIV. Overall, alternative tRNAs (Lys5, Lys6, and Lys1,2) are implicated in initiating RT in humans and macaques, albeit at low levels. Similar to many other biological systems, the virus displays the ability to adapt to suboptimal conditions to allow infection and replication in various hosts.

## Conclusions

Using deep-sequencing analysis, we found that the vast majority of HIV and SIV primer binding site sequences from humans and macaques corresponded to the canonical tRNA^Lys3^, however we did also identify several sequences corresponding to non-canonical tRNAs, including tRNA^Lys5^, tRNA^Lys6^, and tRNA^Lys1,2^ at very low levels. Analysis of the LANL HIV/SIV database also revealed the presence of these non-canonical PBS sequences at very low levels in both SIV and HIV sequences, as well as other PBS mutations that we identified in our deep-sequencing screen which do not correspond to known tRNAs. These data indicate that alternate tRNAs may also contribute to RT initiation, though at much lower frequencies than tRNA^Lys3^. Results from our longitudinal study of wild-type SIVmac239 in macaques demonstrate that the transition from PBS-Lys5 to PBS-Lys3 is rapid and complete with minimal variability over time. These data emphasize the ability of SIV and HIV to adapt to suboptimal conditions and utilize alternate tRNAs to prime RT despite imperfect complementarity.

## Methods

### Animals

Twelves rhesus macaques (*Macaca mulatta*) were housed and cared for in accordance with American Association for Accreditation of Laboratory Animal Care (AAALAC) guidelines in an AAALAC-accredited facility, and all animal procedures were performed according to protocols approved by the Institutional Animal Care and Use Committee of the National Cancer Institute under the standards of the NIH guide for the Care and Use of Laboratory Animals. All animals were free of cercopithecine herpesvirus 1, D-type simian retrovirus, and simian immunodeficiency virus (SIV) at study initiation. Animals were genotyped for common MHC Class 1 alleles such as *Mamu*-A*01, -A*02, -B*08, -B*17 using sequence-specific priming PCR performed as previously described [28]. None of the 12 macaques studied had *Mamu*-A*01, -B*08 and -B*17 alleles. Eight of these rhesus macaques (P014, P031, P032, P034, P038, P051, P058, P088) were infected intrarectally with SIVmac239 (3 × 10^5^ IU) containing the suboptimal PBS sequence and the remaining four animals (ZJ04, ZD28, ZD47, ZD63) were infected intrarectally with SIVmac239Opt (9 × 10^5^ IU) containing the optimal PBS [13]. Blood draws were frequently performed and plasma from various time points were used for PBS sequencing.

### HIV-1 infected individuals

Up to 2 mL of plasma was drawn from 15 HIV-1 infected, but cART untreated, individuals. Donors were predominately male with a mean viral load at time of sample of 3 × 10^4^ vRNA copies/mL enrolled in natural history studies of HIV infection (NIH protocols 95-I-0072 and 00-I-0110) at the NIH Clinical Center. All plasma samples were collected between 2000 and 2015. Two participants (W756 and W905) were infected less than 6 months prior to sampling and the remaining 13 participants were infected for at least 1 year. All participants provided written informed consent for this research.

### RNA isolation and amplification

Viral RNA from plasma was isolated using QIAamp Viral RNA Mini kit (Qiagen) per manufacturer’s protocol and eluted with 60 µL of AVE buffer. Viral RNA was immediately subjected to gene-specific cDNA synthesis with Superscript III reverse transcriptase (ThermoFisher Scientific). Briefly, each cDNA reaction included 1X reverse transcription (RT) buffer, 0.5 mM each deoxynucleoside triphosphate, 5 mM DTT, 2 units/uL RNaseOUT (RNase inhibitor), 10 units/uL SuperScript III reverse transcriptase, and 0.25 uM antisense primer SIV.PBS.R1 5′-CGCCCTTACTGCCTTCACTCAGC-3′ (SIVmac239 position 870-892) for SIV and HIV.PBS.cDNA 5′-CAGCAAGCCGAGTCCTGC-3′ (HXB2 position 691-708) for HIV. The mixture was incubated at 50 °C for 60 min, followed by an increase in temperature to 55 °C for an additional 60 min. The reaction was then heat-inactivated at 70 °C for 15 min and then treated with RNaseH at 37 °C for 20 min. The newly synthesized cDNA was used immediately for PCR and sequencing.

### Illumina sequencing of viral RNA

After cDNA quantification and amplification, samples were prepared for Miseq Illumina sequencing as previously described in [29]. PCR was used to amplify the cDNA and simultaneously add MiSeq adaptors directly onto the amplicon. Reactions were prepared using High Fidelity Platinum Taq per the manufacturer’s instructions (ThermoFisher Scientific), using the SIV forward primer SIV.PBS.P5F 5′-CAG CTA GTG TGT GTT CCC ATC TCT CCTA-3′ (SIVmac239 position 694-721) and SIV reverse primer SIV.PBS.P7R 5′-CGC CCT TAC TGC CTT CAC TCA GC-3′ (SIVmac239 position 907-929) or the HIV forward primer HIV.PBS.P5F 5′-TCT GGT AAC TAG AGA TCC CTC-3′ (HXB2 position 580-600) and HIV reverse HIV.PBS.P7R 5′-CGA GTC CTG CGT CGA GAG-3′ (HXB2 position 683-700) which contain either the F5 or F7 Illumina adaptor sequence and a unique 8 nucleotide index sequence that allows for multiplexing. Up to 10 samples were multiplexed per MiSeq run. Reaction conditions used are as follows: 94 °C, 2 min; 40 × [94 °C, 15 s; 60 °C, 1:30 min; 68 °C, 30 s]; 68 °C, 5 min.

Following PCR, 10 uL from each reaction were pooled and purified using the QIAquick PCR purification kit. The resulting eluted DNA was quantified using the QuBit. The combined DNA sample was diluted to 3.0 nM and 5 uL of this diluted sample was placed in a new tube and denatured with 5 uL 0.2 N NaOH. This sample was vortexed and centrifuged at 280×*g* for 1 min. The sample was incubated at room temperature for 5 min, and 990 uL of chilled HT1 buffer added. This sample was then diluted to 12.5 pM. The control PhiX library was treated similarly. 2 uL of the PhiX library was combined with 3uL Tris-HCl pH 8.5, 0.1% tween-20. 5 uL of 0.2 N HCl was added to the library, and the sample vortexed and centrifuged at 280×*g* for 1 min. The sample was incubated at room temperature for 5 min, and 990 uL of chilled HT1 buffer added. Multiplexed samples and PhiX library were then loaded on the MiSeq reagent tray, and the run initiated.

### Sequence analysis

After the Miseq runs were complete, Fastq files were exported for bioinformatic analyses. Raw reads of each amplicon were deconvoluted and aligned to the PBS sequence. 11 total MiSeq runs were performed with up to 10 samples per run. The average pass-filter sequence reads per run were 22 million. Sequences below 1/10,000 (the lowest template input sample analyzed) were not graphed and represent the lower-limit of detection. Our lowest sample coverage was 6× at the limit of detection (0.0001) for one sample with 54,550 pass filter reads. All other samples had output sequence coverage much greater than this with the highest reads at ~ 3 million sequences per sample generating 500× coverage at the cutoff threshold. Sequences are deposited at NCBI BioProject as PRJNA529689.

## Additional file

**Additional file 1: Table** **S1.** Results of searching the LANL HIV database for all detected PBS variations. Sequences highlighted in blue correspond to HIV-1, sequences in green correspond to SIVmac sequences, and sequences in orange correspond to other natural-host SIV sequences.
