# Supplementary material for: Low-level alternative tRNA priming of reverse transcription of HIV-1 and SIV in vivo
Source: Retrovirology. 2019 Apr 4;16:11. doi: 10.1186/s12977-019-0473-2 (PMC6450007; doi:10.1186/s12977-019-0473-2)
Supplement: Supplementary file 1 — Additional file 1: Table S1. Results of searching the LANL HIV database for all detected PBS variations. Sequences highlighted in blue correspond to HIV-1, sequences in green correspond to SIVmac sequences, and sequences in orange correspond to other natural-host SIV sequences. [file 12977_2019_473_MOESM1_ESM.docx]

| **Lys5** | Accession | Name | Subtype | Country | Year |
| --- | --- | --- | --- | --- | --- |
|  | [JN613611](https://www.hiv.lanl.gov/components/sequence/HIV/asearch/query_one.comp?se_id=462804&accession=JN613611) | 02LGH10_3449s | B | KR | 2002 |
|  | [DQ396399](https://www.hiv.lanl.gov/components/sequence/HIV/asearch/query_one.comp?se_id=102626&accession=DQ396399) | 03ZAPS152MB1 | C | ZA | 2003 |
|  | [EU047655](https://www.hiv.lanl.gov/components/sequence/HIV/asearch/query_one.comp?se_id=42183&accession=EU047655) | 04HJY8_3308 | B | KR | 2004 |
|  | [EF370351](https://www.hiv.lanl.gov/components/sequence/HIV/asearch/query_one.comp?se_id=18300&accession=EF370351) | 05LHO6_2261 | B | KR | 2005 |
|  | [DQ369994](https://www.hiv.lanl.gov/components/sequence/HIV/asearch/query_one.comp?se_id=90693&accession=DQ369994) | 05ZASK247B1 | C | ZA | 2005 |
|  | [KC156198](https://www.hiv.lanl.gov/components/sequence/HIV/asearch/query_one.comp?se_id=526254&accession=KC156198) | T125_2139.gp3 | C | IN | 2009 |
|  | [KF527172](https://www.hiv.lanl.gov/components/sequence/HIV/asearch/query_one.comp?se_id=554574&accession=KF527172) | 4403bmLwk4_fl11 | C | MW | 2008 |
|  | [JF320043](https://www.hiv.lanl.gov/components/sequence/HIV/asearch/query_one.comp?se_id=394441&accession=JF320043) | 502_1400_FL02 | B | US | 2005 |
|  | [MG196913](https://www.hiv.lanl.gov/components/sequence/HIV/asearch/query_one.comp?se_id=817845&accession=MG196913) | 505_1012a.WG02 | B | US | 2012 |
|  | [MG197126](https://www.hiv.lanl.gov/components/sequence/HIV/asearch/query_one.comp?se_id=817632&accession=MG197126) | 505_1958a.WG07 | B | US | 2013 |
|  | [MG197203](https://www.hiv.lanl.gov/components/sequence/HIV/asearch/query_one.comp?se_id=817555&accession=MG197203) | 505_2474a.WG09 | B | US | 2012 |
|  | [JQ779160](https://www.hiv.lanl.gov/components/sequence/HIV/asearch/query_one.comp?se_id=487988&accession=JQ779160) | 707010457.p20 | C | TZ | 2008 |
|  | [KY612729](https://www.hiv.lanl.gov/components/sequence/HIV/asearch/query_one.comp?se_id=756139&accession=KY612729) | DONOR2.A2.5.P6E23 | B | US | 2015 |
|  | [AY535659](https://www.hiv.lanl.gov/components/sequence/HIV/asearch/query_one.comp?se_id=131409&accession=AY535659) | EE0359 | 06_cpx | EE | 2001 |
|  | [JX112859](https://www.hiv.lanl.gov/components/sequence/HIV/asearch/query_one.comp?se_id=553612&accession=JX112859) | TJ070003 | 01_AE | CN | 2007 |
|  | [U67768](https://www.hiv.lanl.gov/components/sequence/HIV/asearch/query_one.comp?se_id=233576&accession=U67768) | TW356 | U | TW | - |
|  | [JN024211](https://www.hiv.lanl.gov/components/sequence/HIV/asearch/query_one.comp?se_id=467141&accession=JN024211) | USPI71101EI7y04051pcWG2B10 | B | US | 2004 |
|  | [JN024200](https://www.hiv.lanl.gov/components/sequence/HIV/asearch/query_one.comp?se_id=466825&accession=JN024200) | USPI83747EI350y05111pcWG2B37 | B | US | 2005 |
|  | [HM583613](https://www.hiv.lanl.gov/components/sequence/HIV/asearch/query_one.comp?se_id=371586&accession=HM583613) | Vin12 | A6 | UA | 2009 |
|  | [KR820322](https://www.hiv.lanl.gov/components/sequence/HIV/asearch/query_one.comp?se_id=647640&accession=KR820322) | Z331M_18Apr09_PL_NFLG_SGA9 | C | ZM | 2009 |
|  | [KR820395](https://www.hiv.lanl.gov/components/sequence/HIV/asearch/query_one.comp?se_id=647567&accession=KR820395) | Z4248F_11Jun10_PL_NFLG_SGA10 | C | ZM | 2010 |
|  | [U80509](https://www.hiv.lanl.gov/components/sequence/HIV/asearch/query_one.comp?se_id=234123&accession=U80509) | LTS2 | B | VE | 1994 |
|  | [AY162225](https://www.hiv.lanl.gov/components/sequence/HIV/asearch/query_one.comp?se_id=151362&accession=AY162225) | TV012 | C | ZA | 1998 |
|  | [AJ006287](https://www.hiv.lanl.gov/components/sequence/HIV/asearch/query_one.comp?se_id=90786&accession=AJ006287) | 89SP061_89ES061 | B | ES | 1989 |
|  | [AB287364](https://www.hiv.lanl.gov/components/sequence/HIV/asearch/query_one.comp?se_id=88818&accession=AB287364) | DR6737 | B | JP | 2005 |
|  | [AB287365](https://www.hiv.lanl.gov/components/sequence/HIV/asearch/query_one.comp?se_id=88817&accession=AB287365) | DR6737 | B | JP | 2005 |
|  | [AB253721](https://www.hiv.lanl.gov/components/sequence/HIV/asearch/query_one.comp?se_id=102986&accession=AB253721) | DR5032 | 01_AE | JP | - |
|  | [AB485652](https://www.hiv.lanl.gov/components/sequence/HIV/asearch/query_one.comp?se_id=312778&accession=AB485652) | ID17 | 01_AE | ID | 1993 |
|  | [AB485657](https://www.hiv.lanl.gov/components/sequence/HIV/asearch/query_one.comp?se_id=312762&accession=AB485657) | BZ163 | F1 | BR | 1990 |
|  | [AB253641](https://www.hiv.lanl.gov/components/sequence/HIV/asearch/query_one.comp?se_id=102618&accession=AB253641) | DR1741 | 01_AE | JP | - |
|  | [AB253664](https://www.hiv.lanl.gov/components/sequence/HIV/asearch/query_one.comp?se_id=102595&accession=AB253664) | DR2594 | 01_AE | JP | - |
|  | [Z11530](https://www.hiv.lanl.gov/components/sequence/HIV/asearch/query_one.comp?se_id=90811&accession=Z11530) | F12CG | B | FR | - |
|  | [JN692452](https://www.hiv.lanl.gov/components/sequence/HIV/asearch/query_one.comp?se_id=451249&accession=JN692452) | 04BR1051 | B | BR | 2004 |
|  | [KT427709](https://www.hiv.lanl.gov/components/sequence/HIV/asearch/query_one.comp?se_id=676718&accession=KT427709) | 10BR_PE072 | B | BR | 2010 |
|  | [M64586](https://www.hiv.lanl.gov/components/sequence/HIV/asearch/query_one.comp?se_id=266807&accession=M64586) | N7201 | - | - | - |
|  | [MF499439](https://www.hiv.lanl.gov/components/sequence/HIV/asearch/query_one.comp?se_id=831489&accession=MF499439) | 700010654.5.d0015.ipe026.5D2 | B | US | 2008 |
|  |  |  |  |  |  |
|  | [X05291](https://www.hiv.lanl.gov/components/sequence/HIV/asearch/query_one.comp?se_id=147335&accession=X05291) | ROD | HIV-2 | SN | 1985 |
|  |  |  |  |  |  |
|  | [M33262](https://www.hiv.lanl.gov/components/sequence/HIV/asearch/query_one.comp?se_id=225804&accession=M33262) | 239 | MAC | US | - |
|  | [AY033146](https://www.hiv.lanl.gov/components/sequence/HIV/asearch/query_one.comp?se_id=208410&accession=AY033146) | 17EFR | MAC | US | - |
|  | [AY033233](https://www.hiv.lanl.gov/components/sequence/HIV/asearch/query_one.comp?se_id=208409&accession=AY033233) | 17EC1 | MAC | US | - |
|  | [D01065](https://www.hiv.lanl.gov/components/sequence/HIV/asearch/query_one.comp?se_id=39196&accession=D01065) | 251_32H_PJ5 | MAC | US | - |
|  |  |  |  |  |  |
|  | [JX860427](https://www.hiv.lanl.gov/components/sequence/HIV/asearch/query_one.comp?se_id=504435&accession=JX860427) | M950 | SMM | US | 2004 |
|  |  |  |  |  |  |
| **Lys1,2** | [L28866](https://www.hiv.lanl.gov/components/sequence/HIV/asearch/query_one.comp?se_id=255147&accession=L28866) | IE-06 | B | US | - |
|  | [MG461324](https://www.hiv.lanl.gov/components/sequence/HIV/asearch/query_one.comp?se_id=841128&accession=MG461324) | 17KYY5-15683s1 | B | KR | 2017 |
|  | [U81466](https://www.hiv.lanl.gov/components/sequence/HIV/asearch/query_one.comp?se_id=245965&accession=U81466) | VE13NEF | B | VE | 1995 |
|  | [KY628215](https://www.hiv.lanl.gov/components/sequence/HIV/asearch/query_one.comp?se_id=784019&accession=KY628215) | BRGO3027 | 90_BF1 | BR | 2007 |
|  | [AF254676](https://www.hiv.lanl.gov/components/sequence/HIV/asearch/query_one.comp?se_id=197369&accession=AF254676) | NT5_3 | A1 | TZ | 1996 |
|  | [AY932713](https://www.hiv.lanl.gov/components/sequence/HIV/asearch/query_one.comp?se_id=108722&accession=AY932713) | TZLM709 | D | TZ | - |
|  | [EU047617](https://www.hiv.lanl.gov/components/sequence/HIV/asearch/query_one.comp?se_id=42221&accession=EU047617) | 95HSH11_3298 | B | KR | 1995 |
|  | [KU168276](https://www.hiv.lanl.gov/components/sequence/HIV/asearch/query_one.comp?se_id=685611&accession=KU168276) | LA22LeRe | F1 | FR | 2004 |
|  |  |  |  |  |  |
|  | [KX089726](https://www.hiv.lanl.gov/components/sequence/HIV/asearch/query_one.comp?se_id=714767&accession=KX089726) | p096.5.p2k12 | MAC | US | - |
|  |  |  |  |  |  |
| **Lys6** | [KX089742](https://www.hiv.lanl.gov/components/sequence/HIV/asearch/query_one.comp?se_id=714751&accession=KX089742) | p097.5.p2g19 | MAC | US | - |
|  |  |  |  |  |  |
| **T1C** | [JN024313](https://www.hiv.lanl.gov/components/sequence/HIV/asearch/query_one.comp?se_id=467090&accession=JN024313) | USPI90770EI99y95101pcWG2B5 | B | US | 1995 |
|  | [KF859740](https://www.hiv.lanl.gov/components/sequence/HIV/asearch/query_one.comp?se_id=577905&accession=KF859740) | DE00210CM013.2 | CRF22 | CM | 2010 |
|  | [GQ999991](https://www.hiv.lanl.gov/components/sequence/HIV/asearch/query_one.comp?se_id=336404&accession=GQ999991) | CAP239_5w_F1 | C | ZA | 2005 |
|  | [JX976672](https://www.hiv.lanl.gov/components/sequence/HIV/asearch/query_one.comp?se_id=522816&accession=JX976672) | C.CAP239.w05.21dps.2_00 | C | ZA | 2005 |
|  |  |  |  |  |  |
|  | [AY965516](https://www.hiv.lanl.gov/components/sequence/HIV/asearch/query_one.comp?se_id=140010&accession=AY965516) | SIVsm77CNPRC_CFU287 | SMM | US | 1977 |
|  | [Y15582](https://www.hiv.lanl.gov/components/sequence/HIV/asearch/query_one.comp?se_id=242308&accession=Y15582) | P037 | SAB | SN | 1991 |
|  |  |  |  |  |  |
| **G2A** | [JN024458](https://www.hiv.lanl.gov/components/sequence/HIV/asearch/query_one.comp?se_id=466696&accession=JN024458) | USPI38051EI3y05121pcWG2B4 | B | US | 2005 |
|  |  |  |  |  |  |
| **G3A** | [JN024559](https://www.hiv.lanl.gov/components/sequence/HIV/asearch/query_one.comp?se_id=466967&accession=JN024559) | USPI11286EI18y05091pcWG2B6 | B | US | 2005 |
|  | [L28858](https://www.hiv.lanl.gov/components/sequence/HIV/asearch/query_one.comp?se_id=255087&accession=L28858) | _1E-10 | B | US | - |
|  |  |  |  |  |  |
| **C4T** | [DQ351232](https://www.hiv.lanl.gov/components/sequence/HIV/asearch/query_one.comp?se_id=102675&accession=DQ351232) | 04ZAPS157MB1 | C | ZA | 2004 |
|  | [EF036531](https://www.hiv.lanl.gov/components/sequence/HIV/asearch/query_one.comp?se_id=77665&accession=EF036531) | Fj062 | 01_AE | CN | 2006 |
|  |  |  |  |  |  |
| **G5A** | [L28904](https://www.hiv.lanl.gov/components/sequence/HIV/asearch/query_one.comp?se_id=255115&accession=L28904) | 4B1LTR | B | US | - |
|  | [DQ396369](https://www.hiv.lanl.gov/components/sequence/HIV/asearch/query_one.comp?se_id=102656&accession=DQ396369) | 03ZAPS123MB1 | C | ZA | 2003 |
|  |  |  |  |  |  |
|  | [KX089651](https://www.hiv.lanl.gov/components/sequence/HIV/asearch/query_one.comp?se_id=714842&accession=KX089651) | PBE.5.A11 | MAC | US | - |
|  |  |  |  |  |  |
| **C6T** | [FJ614916](https://www.hiv.lanl.gov/components/sequence/HIV/asearch/query_one.comp?se_id=268759&accession=FJ614916) | 06LKD11_4096 | 02_AG | KR | 2006 |
|  | [JX447794](https://www.hiv.lanl.gov/components/sequence/HIV/asearch/query_one.comp?se_id=499226&accession=JX447794) | AA093a_LH18 | B | TH | 2006 |
|  |  |  |  |  |  |
| **C7T** | [FM877781](https://www.hiv.lanl.gov/components/sequence/HIV/asearch/query_one.comp?se_id=300911&accession=FM877781) | 02CD_LBTB084 | 26_A5U | CD | 2002 |
|  |  |  |  |  |  |
| **G9A** | [AY921353](https://www.hiv.lanl.gov/components/sequence/HIV/asearch/query_one.comp?se_id=127013&accession=AY921353) | TZMoL47228 | 10_CD | TZ | 2000 |
|  | [MH045880](https://www.hiv.lanl.gov/components/sequence/HIV/asearch/query_one.comp?se_id=818907&accession=MH045880) | DEMB10ES003_2 | 12_BF | ES | 2010 |
|  | [L28874](https://www.hiv.lanl.gov/components/sequence/HIV/asearch/query_one.comp?se_id=255139&accession=L28874) | 2B5 | B | US | - |
|  | [AF197338](https://www.hiv.lanl.gov/components/sequence/HIV/asearch/query_one.comp?se_id=190272&accession=AF197338) | 93TH057 | 01_AE | TH | 1993 |
|  | [EF370239](https://www.hiv.lanl.gov/components/sequence/HIV/asearch/query_one.comp?se_id=18335&accession=EF370239) | 04JWK7_2381s | B | KR | 2004 |
|  |  |  |  |  |  |
| **A10G** | [KY748553](https://www.hiv.lanl.gov/components/sequence/HIV/asearch/query_one.comp?se_id=754760&accession=KY748553) | Donor8.B6_long_J19first | B | US | 2013 |
|  | [EF370292](https://www.hiv.lanl.gov/components/sequence/HIV/asearch/query_one.comp?se_id=18313&accession=EF370292) | 06YJN1_2466s | B | KR | 2006 |
|  | [AF110970](https://www.hiv.lanl.gov/components/sequence/HIV/asearch/query_one.comp?se_id=203563&accession=AF110970) | 96BW1106 | C | BW | 1996 |
|  | [FJ670524](https://www.hiv.lanl.gov/components/sequence/HIV/asearch/query_one.comp?se_id=333757&accession=FJ670524) | X2400-3 | B | ES | 2008 |
|  | [KJ190257](https://www.hiv.lanl.gov/components/sequence/HIV/asearch/query_one.comp?se_id=585850&accession=KJ190257) | R463F_030907_FL55 | A1 | RW | 2007 |
|  | [KJ190254](https://www.hiv.lanl.gov/components/sequence/HIV/asearch/query_one.comp?se_id=585853&accession=KJ190254) | R463F_030907_FL39 | A1 | RW | 2007 |
|  |  |  |  |  |  |
|  | [KX089616](https://www.hiv.lanl.gov/components/sequence/HIV/asearch/query_one.comp?se_id=714877&accession=KX089616) | 7v5.F1 | MAC | US | - |
|  | [KX089728](https://www.hiv.lanl.gov/components/sequence/HIV/asearch/query_one.comp?se_id=714765&accession=KX089728) | p096.5.p2l14 | MAC | US | - |
|  |  |  |  |  |  |
|  | [JX860415](https://www.hiv.lanl.gov/components/sequence/HIV/asearch/query_one.comp?se_id=504441&accession=JX860415) | G078 | SMM | US | 2004 |
|  | [AF301156](https://www.hiv.lanl.gov/components/sequence/HIV/asearch/query_one.comp?se_id=214948&accession=AF301156) | CGU1 | COL | CM | - |
|  |  |  |  |  |  |
| **A11G** | [GQ999976](https://www.hiv.lanl.gov/components/sequence/HIV/asearch/query_one.comp?se_id=336419&accession=GQ999976) | CAP63_5w_F4 | C | ZA | 2005 |
|  | [KY057550](https://www.hiv.lanl.gov/components/sequence/HIV/asearch/query_one.comp?se_id=747393&accession=KY057550) | BVP4tCD4Rep2A.F5.p6j21 | B | US | 2015 |
|  | [AF110971](https://www.hiv.lanl.gov/components/sequence/HIV/asearch/query_one.comp?se_id=203562&accession=AF110971) | 96BW11B01 | C | BW | 1996 |
|  | [KY612696](https://www.hiv.lanl.gov/components/sequence/HIV/asearch/query_one.comp?se_id=756172&accession=KY612696) | DONOR3tCD4Rep2A.F5.p6j21 | B | US | 2015 |
|  | [DQ351233](https://www.hiv.lanl.gov/components/sequence/HIV/asearch/query_one.comp?se_id=102674&accession=DQ351233) | 03ZASK016MB2 | C | ZA | 2003 |
|  | [JN024525](https://www.hiv.lanl.gov/components/sequence/HIV/asearch/query_one.comp?se_id=466984&accession=JN024525) | USPI83747EI210y05071pcWG2B11 | B | US | 2005 |
|  | [L28860](https://www.hiv.lanl.gov/components/sequence/HIV/asearch/query_one.comp?se_id=255085&accession=L28860) | _1E12 | B | US | - |
|  |  |  |  |  |  |
| **C12T** | [KP718920](https://www.hiv.lanl.gov/components/sequence/HIV/asearch/query_one.comp?se_id=643069&accession=KP718920) | 469-66 | U | CM | 2007 |
|  | [KP718917](https://www.hiv.lanl.gov/components/sequence/HIV/asearch/query_one.comp?se_id=643072&accession=KP718917) | 1130-39 | 37_cpx | CM | 2006 |
|  | [KP718918](https://www.hiv.lanl.gov/components/sequence/HIV/asearch/query_one.comp?se_id=643071&accession=KP718918) | 46-10 | A1 | CM | 2007 |
|  |  |  |  |  |  |
| **A13G** | [AB098330](https://www.hiv.lanl.gov/components/sequence/HIV/asearch/query_one.comp?se_id=176670&accession=AB098330) | UG031 | A1 | UG | - |
|  | [AY074891](https://www.hiv.lanl.gov/components/sequence/HIV/asearch/query_one.comp?se_id=89439&accession=AY074891) | 00BWMO35.1 | CD | BW | 2000 |
|  | [KY989957](https://www.hiv.lanl.gov/components/sequence/HIV/asearch/query_one.comp?se_id=775661&accession=KY989957) | 100601 | F1 | ES | 2015 |
|  | [AF443098](https://www.hiv.lanl.gov/components/sequence/HIV/asearch/query_one.comp?se_id=195852&accession=AF443098) | 00BW18113 | C | BW | 2000 |
|  | [AY932697](https://www.hiv.lanl.gov/components/sequence/HIV/asearch/query_one.comp?se_id=108738&accession=AY932697) | TZLM652 | D | TZ | - |
|  | [FJ614995](https://www.hiv.lanl.gov/components/sequence/HIV/asearch/query_one.comp?se_id=268680&accession=FJ614995) | 04HAS1_4194 | 02_AG | KR | 2004 |
|  | [GQ429822](https://www.hiv.lanl.gov/components/sequence/HIV/asearch/query_one.comp?se_id=320339&accession=GQ429822) | clone_6 | A1 | KE | 1995 |
|  |  |  |  |  |  |
|  | [FR751162](https://www.hiv.lanl.gov/components/sequence/HIV/asearch/query_one.comp?se_id=400958&accession=FR751162) | SIVsunK08_Gab | SUN | GA | 2008 |
|  |  |  |  |  |  |
| **G14A** | [DQ164125](https://www.hiv.lanl.gov/components/sequence/HIV/asearch/query_one.comp?se_id=102720&accession=DQ164125) | 04ZAPS187B1 | C | ZA | 2004 |
|  | [KJ849794](https://www.hiv.lanl.gov/components/sequence/HIV/asearch/query_one.comp?se_id=607382&accession=KJ849794) | 10BR_SP017 | BF1 | BR | 2010 |
|  |  |  |  |  |  |
| **G15A** | [EU047607](https://www.hiv.lanl.gov/components/sequence/HIV/asearch/query_one.comp?se_id=42231&accession=EU047607) | 03CSR3_3089 | B | KR | 2003 |
|  | [DQ853454](https://www.hiv.lanl.gov/components/sequence/HIV/asearch/query_one.comp?se_id=100524&accession=DQ853454) | 14295_1 | B | US | 2000 |
|  | [KC156476](https://www.hiv.lanl.gov/components/sequence/HIV/asearch/query_one.comp?se_id=526115&accession=KC156476) | 702010141_CH141.w12.gp20 | C | MW | 2007 |
|  | [AF254675](https://www.hiv.lanl.gov/components/sequence/HIV/asearch/query_one.comp?se_id=197370&accession=AF254675) | NT4_3 | A1 | TZ | 1996 |
|  | [GQ999982](https://www.hiv.lanl.gov/components/sequence/HIV/asearch/query_one.comp?se_id=336413&accession=GQ999982) | CAP206_8w_F1 | C | ZA | 2005 |
|  |  |  |  |  |  |
| **G16A** | **NO MATCHES** |  |  |  |  |
|  |  |  |  |  |  |
| **A17G** | [JN024398](https://www.hiv.lanl.gov/components/sequence/HIV/asearch/query_one.comp?se_id=466726&accession=JN024398) | USPI38417EI112y05081pcWG2B26 | B | US | 2005 |
|  | [JQ779120](https://www.hiv.lanl.gov/components/sequence/HIV/asearch/query_one.comp?se_id=488008&accession=JQ779120) | 703010256.gp3 | C | MW | 2009 |
|  | [AY772690](https://www.hiv.lanl.gov/components/sequence/HIV/asearch/query_one.comp?se_id=102927&accession=AY772690) | SK023B2 | C | ZA | 2003 |
|  | [JQ316131](https://www.hiv.lanl.gov/components/sequence/HIV/asearch/query_one.comp?se_id=477597&accession=JQ316131) | 03HJY8 | B | KR | 2003 |
|  | [JN024555](https://www.hiv.lanl.gov/components/sequence/HIV/asearch/query_one.comp?se_id=466969&accession=JN024555) | USPI38417EI243y05121pcWG2B13 | B | US | 2005 |
|  | [JN024466](https://www.hiv.lanl.gov/components/sequence/HIV/asearch/query_one.comp?se_id=466692&accession=JN024466) | USPI51550EI3285y05051pcWG2B8 | B | US | 2005 |
|  | [AY932666](https://www.hiv.lanl.gov/components/sequence/HIV/asearch/query_one.comp?se_id=108769&accession=AY932666) | TZLM624 | A1 | TZ | - |
|  | [AB052607](https://www.hiv.lanl.gov/components/sequence/HIV/asearch/query_one.comp?se_id=64034&accession=AB052607) | 98TNIH054 | 01_AE | TH | 1998 |
|  | [DQ275646](https://www.hiv.lanl.gov/components/sequence/HIV/asearch/query_one.comp?se_id=102711&accession=DQ275646) | 03ZAPS133MB1 | C | ZA | 2003 |
|  | [AF443099](https://www.hiv.lanl.gov/components/sequence/HIV/asearch/query_one.comp?se_id=195851&accession=AF443099) | 00BW18595_5 | C | BW | 2000 |
|  | [HM067748](https://www.hiv.lanl.gov/components/sequence/HIV/asearch/query_one.comp?se_id=353600&accession=HM067748) | nx2 | 08_BC | CN | 2006 |
|  |  |  |  |  |  |
|  | [EF070329](https://www.hiv.lanl.gov/components/sequence/HIV/asearch/query_one.comp?se_id=78646&accession=EF070329) | CM1246 | MUS-2 | CM | 2001 |
|  | [AF468658](https://www.hiv.lanl.gov/components/sequence/HIV/asearch/query_one.comp?se_id=192557&accession=AF468658) | CN71 | GSN | CM | 1999 |
|  |  |  |  |  |  |
| **C18T** | [AF196766](https://www.hiv.lanl.gov/components/sequence/HIV/asearch/query_one.comp?se_id=223944&accession=AF196766) | 96GH22 | AU | GH | 1996 |
|  |  |  |  |  |  |
| **T1G** | [EU047678](https://www.hiv.lanl.gov/components/sequence/HIV/asearch/query_one.comp?se_id=42160&accession=EU047678) | 07YJN2_3211 | B | KR | 2007 |
|  | [JN417239](https://www.hiv.lanl.gov/components/sequence/HIV/asearch/query_one.comp?se_id=490080&accession=JN417239) | 33365 | 50_A1D | GB | 2003 |
|  | [GQ432647](https://www.hiv.lanl.gov/components/sequence/HIV/asearch/query_one.comp?se_id=317514&accession=GQ432647) | clone_2783 | A1 | KE | 1998 |
|  | [EF036534](https://www.hiv.lanl.gov/components/sequence/HIV/asearch/query_one.comp?se_id=77662&accession=EF036534) | Fj065 | 01_AE | CN | 2005 |
|  | [EU938092](https://www.hiv.lanl.gov/components/sequence/HIV/asearch/query_one.comp?se_id=9292&accession=EU938092) | 97RU207 | A6 | RU | 1997 |
|  |  |  |  |  |  |
|  | [AY965498](https://www.hiv.lanl.gov/components/sequence/HIV/asearch/query_one.comp?se_id=140028&accession=AY965498) | SIVsm02YNPRC_FAL | SMM | US | 2002 |
|  |  |  |  |  |  |
| **T1A** | [KP718922](https://www.hiv.lanl.gov/components/sequence/HIV/asearch/query_one.comp?se_id=643067&accession=KP718922) | 663-13 | 0102A | CM | 2007 |
|  | [KY778265](https://www.hiv.lanl.gov/components/sequence/HIV/asearch/query_one.comp?se_id=794475&accession=KY778265) | 2026_N_1 | B | US | 2013 |
|  |  |  |  |  |  |
| **G2T** | **NO MATCHES** |  |  |  |  |
|  |  |  |  |  |  |
| **G3T** | [AY610965](https://www.hiv.lanl.gov/components/sequence/HIV/asearch/query_one.comp?se_id=123366&accession=AY610965) | 98SP7030 | 11_cpx | ES | 1998 |
|  |  |  |  |  |  |
| **C4A** | [KY057473](https://www.hiv.lanl.gov/components/sequence/HIV/asearch/query_one.comp?se_id=747470&accession=KY057473) | BVP12rCD4Rep2.3.F8.p5f17 | B | US | 2015 |
|  | [MF499548](https://www.hiv.lanl.gov/components/sequence/HIV/asearch/query_one.comp?se_id=833917&accession=MF499548) | 700010654.5.d0026.ipe026.5G11 | B | US | 2008 |
|  | [KY553107](https://www.hiv.lanl.gov/components/sequence/HIV/asearch/query_one.comp?se_id=756673&accession=KY553107) | DONOR1rCD4Rep2.3.F8.p5f17 | B | US | 2015 |
|  |  |  |  |  |  |
| **C4G** | **NO MATCHES** |  |  |  |  |
|  |  |  |  |  |  |
| **G5T** | **NO MATCHES** |  |  |  |  |
|  |  |  |  |  |  |
| **C7G** | [DQ358810](https://www.hiv.lanl.gov/components/sequence/HIV/asearch/query_one.comp?se_id=89382&accession=DQ358810) | 02BR013 | B | BR | 2002 |
|  |  |  |  |  |  |
| **C8A** | [EF370360](https://www.hiv.lanl.gov/components/sequence/HIV/asearch/query_one.comp?se_id=18298&accession=EF370360) | 05YGS8_2623 | B | KR | 2005 |
|  |  |  |  |  |  |
| **G9T** | **NO MATCHES** |  |  |  |  |
|  |  |  |  |  |  |
| **A10T** | **NO MATCHES** |  |  |  |  |
|  |  |  |  |  |  |
| **A10C** | [EF370283](https://www.hiv.lanl.gov/components/sequence/HIV/asearch/query_one.comp?se_id=18318&accession=EF370283) | 01YJN9_2503s1 | B | KR | 2001 |
|  |  |  |  |  |  |
| **A11T** | **NO MATCHES** |  |  |  |  |
|  |  |  |  |  |  |
| **A11C** | [KF011493](https://www.hiv.lanl.gov/components/sequence/HIV/asearch/query_one.comp?se_id=548430&accession=KF011493) | JL100091 | BC | CN | 2010 |
|  | [DQ056416](https://www.hiv.lanl.gov/components/sequence/HIV/asearch/query_one.comp?se_id=102767&accession=DQ056416) | 04ZASK176B1 | C | ZA | 2004 |
|  | [AF443090](https://www.hiv.lanl.gov/components/sequence/HIV/asearch/query_one.comp?se_id=195860&accession=AF443090) | 00BW087421 | C | BW | 2000 |
|  | [AF443105](https://www.hiv.lanl.gov/components/sequence/HIV/asearch/query_one.comp?se_id=195845&accession=AF443105) | 00BW2127214 | C | BW | 2000 |
|  |  |  |  |  |  |
| **A13T** | [MG196817](https://www.hiv.lanl.gov/components/sequence/HIV/asearch/query_one.comp?se_id=817941&accession=MG196817) | 505_0772a.WG4 | B | US | 2012 |
|  |  |  |  |  |  |
| **A13C** | [JN024343](https://www.hiv.lanl.gov/components/sequence/HIV/asearch/query_one.comp?se_id=467075&accession=JN024343) | USPI88403EI14y05121pcWG2B4 | B | US | 2005 |
|  | [EF370254](https://www.hiv.lanl.gov/components/sequence/HIV/asearch/query_one.comp?se_id=18247&accession=EF370254) | 04CWS5_2698 | B | KR | 2004 |
|  |  |  |  |  |  |
| **G14T** | **NO MATCHES** |  |  |  |  |
|  |  |  |  |  |  |
| **G15T** | **NO MATCHES** |  |  |  |  |
|  |  |  |  |  |  |
| **A17T** | [AF538306](https://www.hiv.lanl.gov/components/sequence/HIV/asearch/query_one.comp?se_id=187797&accession=AF538306) | C76 | B | AU | 1995 |
|  | [K03458](https://www.hiv.lanl.gov/components/sequence/HIV/asearch/query_one.comp?se_id=256006&accession=K03458) | Z6_Z2Z6_Z34 | D | CD | 1985 |
|  |  |  |  |  |  |
| **A17C** | [FJ614984](https://www.hiv.lanl.gov/components/sequence/HIV/asearch/query_one.comp?se_id=268691&accession=FJ614984) | 05KSN5_4230 | 02_AG | KR | 2005 |
|  | [EU938119](https://www.hiv.lanl.gov/components/sequence/HIV/asearch/query_one.comp?se_id=9265&accession=EU938119) | 04RU1139 | A6 | RU | 2004 |
|  | [AF443108](https://www.hiv.lanl.gov/components/sequence/HIV/asearch/query_one.comp?se_id=195842&accession=AF443108) | 00BW38193 | C | BW | 2000 |
|  |  |  |  |  |  |
| **C18G** | [KX505408](https://www.hiv.lanl.gov/components/sequence/HIV/asearch/query_one.comp?se_id=715711&accession=KX505408) | 2286 | B | US | 2014 |
|  | [EF370317](https://www.hiv.lanl.gov/components/sequence/HIV/asearch/query_one.comp?se_id=18208&accession=EF370317) | 03KGS1_2382 | B | KR | 2003 |
|  |  |  |  |  |  |
|  | [Y15576](https://www.hiv.lanl.gov/components/sequence/HIV/asearch/query_one.comp?se_id=242314&accession=Y15576) | G022 | SAB | SN | 1991 |
|  | [Y15575](https://www.hiv.lanl.gov/components/sequence/HIV/asearch/query_one.comp?se_id=242315&accession=Y15575) | G021 | SAB | SN | 1991 |
|  |  |  |  |  |  |
| **C14A/G5A** | [GQ999986](https://www.hiv.lanl.gov/components/sequence/HIV/asearch/query_one.comp?se_id=336409&accession=GQ999986) | CAP244_8w_F1 | C | ZA | 2005 |
|  | [JN024410](https://www.hiv.lanl.gov/components/sequence/HIV/asearch/query_one.comp?se_id=466720&accession=JN024410) | USPI38417EI140y05091pcWG2B9 | B | US | 2005 |
|  |  |  |  |  |  |
| **A10G/A13G** | **NO MATCHES** |  |  |  |  |
|  |  |  |  |  |  |
| **A13G/G14T** | **NO MATCHES** |  |  |  |  |
|  |  |  |  |  |  |
| **G16A/C18G** | **NO MATCHES** |  |  |  |  |
|  |  |  |  |  |  |
| **G16A/A17C** | [AY610978](https://www.hiv.lanl.gov/components/sequence/HIV/asearch/query_one.comp?se_id=123353&accession=AY610978) | 03SP91571 | 11_cpx | ES | 2003 |
|  | [AY452652](https://www.hiv.lanl.gov/components/sequence/HIV/asearch/query_one.comp?se_id=167252&accession=AY452652) | PHD74H4 | C | ET | 1997 |
|  | [AY452651](https://www.hiv.lanl.gov/components/sequence/HIV/asearch/query_one.comp?se_id=167253&accession=AY452651) | PHD74D3 | C | ET | 1997 |
|  |  |  |  |  |  |
| **G16A/A17C/C18T** | [L39106](https://www.hiv.lanl.gov/components/sequence/HIV/asearch/query_one.comp?se_id=L39106) | IBNG | 02_AG | NG | - |
|  | [M13136](https://www.hiv.lanl.gov/components/sequence/HIV/asearch/query_one.comp?se_id=266801&accession=M13136) | CDC451-a | B | US | 1984 |
|  |  |  |  |  |  |
|  | [E02677](https://www.hiv.lanl.gov/components/sequence/HIV/asearch/query_one.comp?se_id=116188&accession=E02677) | GB1 | MND-1 | GB | - |
|  | [M27470](https://www.hiv.lanl.gov/components/sequence/HIV/asearch/query_one.comp?se_id=198176&accession=M27470) | MNDGB1 | MND-1 | GA | - |
|  |  |  |  |  |  |
|  | [M19499](https://www.hiv.lanl.gov/components/sequence/HIV/asearch/query_one.comp?se_id=217039&accession=M19499) | 251_BK28 | MAC | US | - |
|  |  |  |  |  |  |
| **G9A/A10C/A11C** | **NO MATCHES** |  |  |  |  |
|  |  |  |  |  |  |
| **G15A/A17T/C18G** | **NO MATCHES** |  |  |  |  |
|  |  |  |  |  |  |
| **Lys1,2 A9G** | **NO MATCHES** |  |  |  |  |
|  |  |  |  |  |  |
